# *rac*-*cis*-Dicarbonyl­chlorido{1-[2-(diphenyl­phosphanyl-κ*P*)benz­yl]-3-(phenyl-κ*C* ^1^)imidazol-2-yl­idene-κ*C* ^2^}ruthenium(II) dichloro­methane monosolvate

**DOI:** 10.1107/S1600536812036665

**Published:** 2012-08-31

**Authors:** Gregory J. Domski, Wiktoria H. Pecak, Dale C. Swenson

**Affiliations:** a Augustana College, Department of Chemistry, 639 38th Street, Rock Island, IL 61201, USA; b The University of Iowa, W437 Chemistry Building, Iowa City, IA 52242-1294, USA

## Abstract

In the title compound, [Ru(C_28_H_22_N_2_P)Cl(CO)_2_]·CH_2_Cl_2_, the Ru^II^ atom exhibits a distorted octa­hedral coordination geometry. The *N*-phenyl group of the ligand has undergone orthometalation; as a result, the tridentate phosphane-functionalized *N*-heterocyclic carbene ligand is coordinating in a meridional fashion. This complex is of inter­est with respect to transfer hydrogenation catalysis and also provides an example of C—H activation behavior in late transition metal complexes. The dichloro­methane solvent mol­ecule is disordered over two sets of sites with an occupancy ratio of 0.873 (14):0.127 (14).

## Related literature

For a review of transition metal catalysts supported by donor-functionalized *N*-heterocyclic carbenes, see: Cavell & Normand (2008 ▶). For the first reported synthesis of the imidazolium chloride pro-ligand, see: Wang *et al.* (2005 ▶). For the structure of a similar mol­ecule bearing an *N*-mesityl moiety that has not undergone orthometalation, see: Domski *et al.* (2012 ▶).
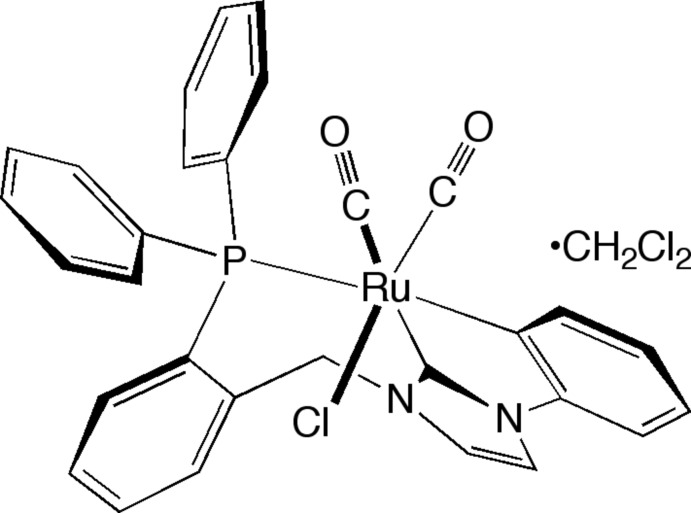


## Experimental

### 

#### Crystal data

[Ru(C_28_H_22_N_2_P)Cl(CO)_2_]·CH_2_Cl_2_
*M* *_r_* = 694.91
Triclinic, 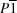

*a* = 8.1600 (9) Å
*b* = 12.1940 (13) Å
*c* = 14.9713 (16) Å
α = 100.109 (5)°
β = 93.560 (5)°
γ = 93.469 (5)°
*V* = 1459.9 (3) Å^3^
*Z* = 2
Mo *K*α radiation
μ = 0.90 mm^−1^
*T* = 210 K
0.17 × 0.08 × 0.03 mm

#### Data collection

Nonius KappaCCD diffractometer
Absorption correction: multi-scan (*HKL* *SCALEPACK*; Otwinowski & Minor, 1997 ▶) *T*_min_ = 0.862, *T*_max_ = 0.974
20414 measured reflections
5349 independent reflections
4214 reflections with *I* > 2σ(*I*)
*R*_int_ = 0.038

#### Refinement

*R*[*F* ^2^ > 2σ(*F* ^2^)] = 0.036
*wR*(*F* ^2^) = 0.077
*S* = 1.02
5349 reflections
383 parameters
22 restraints
H-atom parameters constrained
Δρ_max_ = 0.60 e Å^−3^
Δρ_min_ = −0.45 e Å^−3^

### 

Data collection: *COLLECT* (Nonius, 2000) ▶; cell refinement: *SCALEPACK* (Otwinowski & Minor, 1997 ▶); data reduction: *DENZO* (Otwinowski & Minor, 1997 ▶) and *SCALEPACK*; program(s) used to solve structure: *SHELXTL* (Sheldrick, 2008 ▶); program(s) used to refine structure: *SHELXTL*; molecular graphics: *SHELXTL*; software used to prepare material for publication: *SHELXTL*.

## Supplementary Material

Crystal structure: contains datablock(s) I, global. DOI: 10.1107/S1600536812036665/hp2045sup1.cif

Structure factors: contains datablock(s) I. DOI: 10.1107/S1600536812036665/hp2045Isup2.hkl

Additional supplementary materials:  crystallographic information; 3D view; checkCIF report

## Figures and Tables

**Table 1:** Selected bond lengths (Å)

Ru1—C1	1.860 (4)
Ru1—C2	1.932 (4)
Ru1—C3	2.065 (3)
Ru1—C11	2.129 (3)
Ru1—P1	2.4265 (9)
Ru1—Cl1	2.4737 (9)

**Fig. 1.**
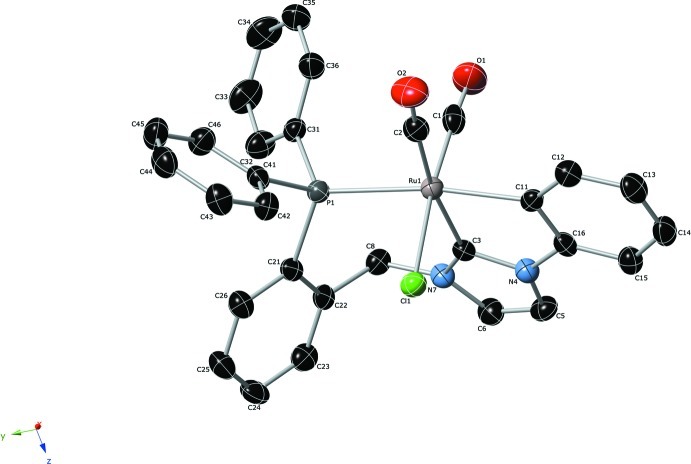
The molecular structure of the title complex with ellipsoids drawn at the 50% probability level. Hydrogen atoms and a dichloromethane molecule of crystallization were omitted for clarity.

## supplementary crystallographic information

### Comment

Donor-functionalized *N*-heterocyclic carbenes (NHCs) are employed as ancillary ligands for catalytically active transition metals since they have been shown to prevent common decomposition pathways (*e.g.* reductive elimination) (Cavell & Normand, 2008). Our research has focused on preparing novel ruthenium(II) complexes supported by donor-functionalized NHCs. In particular we have focused on phosphine-functionalized NHCs since both of these moieties are strong σ-donors and have been shown to effectively stabilize ruthenium(II) at catalytically relevant (*i.e.* elevated) temperatures. The title compound was exceptionally difficult to characterize *via*^1^H and ^13^C{^1^H} NMR spectroscopy due to extensive overlapping of peaks in the aromatic regions. In order to establish the three-dimensional structure of this molecule we grew single crystals of the title complex and collected X-ray diffraction data. Upon solving the structure we were surprised to find that the *N*-phenyl moiety of the ligand had undergone orthometalation. While C—H activation by ruthenium(II) is not without precedent, we had not expected this to happen; it would have been difficult to elucidate this behavior without the aid of X-ray crystallography.

In the solid state, the geometry about ruthenium was distorted octahedral with a C(3)–Ru–C(11) bond angle of 77.79 (12)°. The Ru-carbonyl distances were inequivalent with the Ru–C(2) distance being 0.072 (0) Å longer than the Ru–C(1) distance; this observation is consistent with the stronger *trans*-influence of the NHC relative to chloride.

### Experimental

Single crystals suitable for X-ray diffraction studies were grown by vapor diffusion of diethyl ether onto a saturated dichloromethane solution of the title compound.

### Refinement

The preliminary model of the structure was obtained using XS, a direct methods program. Least-squares refining of the model *versus*. the data was performed with XL computer program. Illustrations were made with the *XP* program and tables were made with the XCIF program. All are in the *SHELXTL* v6.1 package. Thermal ellipsoids shown in the illustrations are at the 50% level unless otherwise noted. All non-hydrogen atoms were refined with anisotropic thermal parameters. A disordered dichloromethane molecule is included in the structure. The relative occupancy refined to 0.873 (14):0.127 (14). The two disorder sites were restrained to have the same conformation, Uaniso(C51)=Uaniso(C51'). The anisotropic displacement parameters were restrained with the rigid body(DELU) restraint and the similarity (SIMU) restraint. All H atoms were included with the riding model using the XL program default values. No further restraints or constraints were imposed on the refinement model.

### Crystal data

**Table:**

[Ru(C_28_H_22_N_2_P)Cl(CO)_2_]·CH_2_Cl_2_	*Z* = 2
*M**_r_* = 694.91	*F*(000) = 700
Triclinic, *P*1	*D*_x_ = 1.581 Mg m^−^^3^
Hall symbol: -P 1	Mo *K*α radiation, λ = 0.71073 Å
*a* = 8.1600 (9) Å	Cell parameters from 5183 reflections
*b* = 12.1940 (13) Å	θ = 1.0–27.9°
*c* = 14.9713 (16) Å	µ = 0.90 mm^−^^1^
α = 100.109 (5)°	*T* = 210 K
β = 93.560 (5)°	Plate, colorless
γ = 93.469 (5)°	0.17 × 0.08 × 0.03 mm
*V* = 1459.9 (3) Å^3^	

### Data collection

**Table:**

Nonius KappaCCD diffractometer	5349 independent reflections
Radiation source: fine-focus sealed tube	4214 reflections with *I* > 2σ(*I*)
Graphite monochromator	*R*_int_ = 0.038
Detector resolution: 9 pixels mm^-1^	θ_max_ = 25.5°, θ_min_ = 2.8°
phi and ω scans	*h* = −9→9
Absorption correction: multi-scan (*HKL**SCALEPACK*; Otwinowski & Minor, 1997)	*k* = −14→14
*T*_min_ = 0.862, *T*_max_ = 0.974	*l* = −18→18
20414 measured reflections	

### Refinement

**Table:**

Refinement on *F*^2^	Primary atom site location: structure-invariant direct methods
Least-squares matrix: full	Secondary atom site location: difference Fourier map
*R*[*F*^2^ > 2σ(*F*^2^)] = 0.036	Hydrogen site location: inferred from neighbouring sites
*wR*(*F*^2^) = 0.077	H-atom parameters constrained
*S* = 1.02	*w* = 1/[σ^2^(*F*_o_^2^) + (0.0237*P*)^2^ + 1.1613*P*] where *P* = (*F*_o_^2^ + 2*F*_c_^2^)/3
5349 reflections	(Δ/σ)_max_ = 0.001
383 parameters	Δρ_max_ = 0.60 e Å^−^^3^
22 restraints	Δρ_min_ = −0.45 e Å^−^^3^

### Special details

**Table:**

Experimental. The title compound was prepared by allowing 1-phenyl-3-(2-diphenylphosphinobenzyl)-1*H*-imidazol-3-ium chloride (0.4997 g) (Wang *et al.*, 2005) to react with Ag_2_O (0.2502 g) in dry, degassed dichloromethane under a nitrogen atmosphere in the absence of light; 4 Å molecular sieves (approximately 0.5 g) were added to the mixture at the beginning of the reaction. After 24 h, the reaction mixture was filtered through Celite^TM^ under an atmosphere of nitrogen into a flask containing [Ru(CO)_3_Cl_2_]_2_ (0.1340 g); the reaction mixture was allowed to stir for 24 h in the dark. After stirring overnight, the reaction mixture was filtered through Celite^TM^ under an atmosphere of dry nitrogen and all volatiles were removed *in vacuo*. The solid residue was purified *via* column chromatography (SiO_2_, 40:1 CH_2_Cl_2_/MeOH) to furnish a yellow solid. Single crystals of the title compound were grown by dissolving the crude product in the minimum amount of dichloromethane and allowing diethyl ether to slowly evaporate, condense, and diffuse into the dichloromethane solution.
Geometry. All e.s.d.'s (except the e.s.d. in the dihedral angle between two l.s. planes) are estimated using the full covariance matrix. The cell e.s.d.'s are taken into account individually in the estimation of e.s.d.'s in distances, angles and torsion angles; correlations between e.s.d.'s in cell parameters are only used when they are defined by crystal symmetry. An approximate (isotropic) treatment of cell e.s.d.'s is used for estimating e.s.d.'s involving l.s. planes.
Refinement. Refinement of *F*^2^ against ALL reflections. The weighted *R*-factor *wR* and goodness of fit *S* are based on *F*^2^, conventional *R*-factors *R* are based on *F*, with *F* set to zero for negative *F*^2^. The threshold expression of *F*^2^ > σ(*F*^2^) is used only for calculating *R*-factors(gt) *etc*. and is not relevant to the choice of reflections for refinement. *R*-factors based on *F*^2^ are statistically about twice as large as those based on *F*, and *R*- factors based on ALL data will be even larger.Several low angle reflections were omitted from the final cycles of refinement due to beam-stop shadowing effects.A disordered dichloromethane molecule is included in the structure. The relative occupancy refined to 0.873 (14):0.127 (14). The two disorder sites were restrained to have the same conformation, Uaniso(C41)=Uaniso(C41'). The anisotropic displacement parameters were restrained with the rigid body restraint and the simu restraint.

### Fractional atomic coordinates and isotropic or equivalent isotropic displacement parameters (Å^2^)

**Table:**

	*x*	*y*	*z*	*U*_iso_*/*U*_eq_	Occ. (<1)
Ru1	0.56260 (3)	0.10472 (2)	0.259925 (18)	0.02297 (9)	
Cl1	0.71756 (10)	0.21064 (7)	0.39873 (5)	0.0297 (2)	
C1	0.4406 (4)	0.0144 (3)	0.1624 (3)	0.0310 (8)	
O1	0.3680 (3)	−0.0442 (2)	0.10503 (18)	0.0505 (7)	
C2	0.7517 (4)	0.1111 (3)	0.1890 (2)	0.0300 (8)	
O2	0.8606 (3)	0.1083 (2)	0.14529 (19)	0.0503 (7)	
C3	0.3718 (4)	0.0743 (2)	0.3387 (2)	0.0242 (7)	
N4	0.3856 (3)	−0.0118 (2)	0.38523 (18)	0.0255 (6)	
C5	0.2468 (4)	−0.0284 (3)	0.4310 (2)	0.0306 (8)	
H5	0.2278	−0.0825	0.4675	0.037*	
C6	0.1447 (4)	0.0482 (3)	0.4128 (2)	0.0311 (8)	
H6	0.0399	0.0582	0.4344	0.037*	
N7	0.2224 (3)	0.1101 (2)	0.35618 (18)	0.0247 (6)	
C8	0.1486 (4)	0.1970 (3)	0.3140 (2)	0.0272 (8)	
H8A	0.0305	0.1947	0.3229	0.033*	
H8B	0.1611	0.1811	0.2484	0.033*	
C11	0.6328 (4)	−0.0402 (2)	0.3096 (2)	0.0229 (7)	
C12	0.7679 (4)	−0.1015 (3)	0.2936 (2)	0.0312 (8)	
H12	0.8445	−0.0801	0.2543	0.037*	
C13	0.7943 (4)	−0.1942 (3)	0.3337 (2)	0.0358 (9)	
H13	0.8871	−0.2346	0.3207	0.043*	
C14	0.6857 (4)	−0.2270 (3)	0.3922 (2)	0.0361 (9)	
H14	0.7040	−0.2901	0.4186	0.043*	
C15	0.5489 (4)	−0.1671 (3)	0.4124 (2)	0.0315 (8)	
H15	0.4744	−0.1879	0.4530	0.038*	
C16	0.5262 (4)	−0.0757 (3)	0.3706 (2)	0.0253 (7)	
P1	0.46973 (10)	0.27908 (7)	0.22565 (6)	0.0231 (2)	
C21	0.3631 (4)	0.3606 (2)	0.3170 (2)	0.0233 (7)	
C22	0.2256 (4)	0.3134 (3)	0.3529 (2)	0.0241 (7)	
C23	0.1539 (4)	0.3756 (3)	0.4245 (2)	0.0314 (8)	
H23	0.0611	0.3442	0.4475	0.038*	
C24	0.2149 (4)	0.4827 (3)	0.4629 (2)	0.0354 (9)	
H24	0.1661	0.5227	0.5127	0.043*	
C25	0.3484 (4)	0.5304 (3)	0.4277 (2)	0.0359 (9)	
H25	0.3894	0.6039	0.4524	0.043*	
C26	0.4217 (4)	0.4696 (3)	0.3557 (2)	0.0308 (8)	
H26	0.5131	0.5025	0.3325	0.037*	
C31	0.3271 (4)	0.2636 (3)	0.1239 (2)	0.0248 (7)	
C32	0.1787 (4)	0.3131 (3)	0.1231 (2)	0.0365 (9)	
H32	0.1491	0.3589	0.1761	0.044*	
C33	0.0732 (4)	0.2955 (3)	0.0446 (3)	0.0438 (10)	
H33	−0.0280	0.3286	0.0450	0.053*	
C34	0.1160 (5)	0.2301 (3)	−0.0336 (3)	0.0462 (11)	
H34	0.0436	0.2169	−0.0864	0.055*	
C35	0.2665 (5)	0.1840 (3)	−0.0341 (3)	0.0422 (10)	
H35	0.2982	0.1413	−0.0880	0.051*	
C36	0.3710 (4)	0.2001 (3)	0.0444 (2)	0.0338 (8)	
H36	0.4725	0.1675	0.0435	0.041*	
C41	0.6282 (4)	0.3817 (2)	0.2024 (2)	0.0254 (7)	
C42	0.7775 (4)	0.4017 (3)	0.2551 (2)	0.0308 (8)	
H42	0.7995	0.3589	0.3003	0.037*	
C43	0.8933 (4)	0.4832 (3)	0.2419 (3)	0.0349 (9)	
H43	0.9922	0.4973	0.2790	0.042*	
C44	0.8630 (4)	0.5444 (3)	0.1737 (3)	0.0380 (9)	
H44	0.9428	0.5988	0.1635	0.046*	
C45	0.7169 (5)	0.5257 (3)	0.1210 (3)	0.0412 (10)	
H45	0.6969	0.5679	0.0751	0.049*	
C46	0.5985 (4)	0.4454 (3)	0.1349 (2)	0.0341 (9)	
H46	0.4982	0.4337	0.0990	0.041*	
C51	0.3690 (10)	−0.3020 (15)	0.1999 (6)	0.0623 (17)	0.873 (14)
H51A	0.4006	−0.3649	0.2282	0.075*	0.873 (14)
H51B	0.4636	−0.2468	0.2081	0.075*	0.873 (14)
Cl3	0.3192 (5)	−0.3489 (3)	0.08393 (18)	0.0632 (8)	0.873 (14)
Cl4	0.2056 (3)	−0.2424 (5)	0.2530 (3)	0.0888 (15)	0.873 (14)
C51'	0.367 (7)	−0.296 (11)	0.196 (4)	0.0623 (17)	0.127 (14)
H51C	0.4704	−0.3223	0.2192	0.075*	0.127 (14)
H51D	0.3909	−0.2204	0.1847	0.075*	0.127 (14)
Cl3'	0.294 (4)	−0.384 (3)	0.0949 (13)	0.087 (5)	0.127 (14)
Cl4'	0.226 (3)	−0.293 (3)	0.2777 (13)	0.094 (6)	0.127 (14)

### Atomic displacement parameters (Å^2^)

**Table:**

	*U*^11^	*U*^22^	*U*^33^	*U*^12^	*U*^13^	*U*^23^
Ru1	0.02152 (15)	0.02327 (15)	0.02432 (16)	0.00118 (11)	0.00288 (11)	0.00453 (11)
Cl1	0.0307 (5)	0.0306 (5)	0.0274 (5)	−0.0013 (4)	0.0017 (4)	0.0054 (4)
C1	0.0274 (19)	0.0269 (19)	0.043 (2)	0.0078 (16)	0.0102 (17)	0.0122 (18)
O1	0.0582 (19)	0.0437 (16)	0.0441 (17)	−0.0022 (14)	−0.0087 (15)	−0.0008 (14)
C2	0.030 (2)	0.0283 (19)	0.030 (2)	−0.0018 (16)	−0.0002 (17)	0.0030 (16)
O2	0.0388 (16)	0.0631 (19)	0.0481 (18)	−0.0039 (14)	0.0217 (14)	0.0039 (14)
C3	0.0273 (18)	0.0224 (17)	0.0222 (18)	0.0027 (14)	0.0033 (14)	0.0013 (14)
N4	0.0225 (15)	0.0248 (15)	0.0292 (16)	0.0015 (12)	0.0050 (12)	0.0041 (12)
C5	0.032 (2)	0.0321 (19)	0.028 (2)	−0.0039 (16)	0.0085 (16)	0.0078 (16)
C6	0.0265 (19)	0.0314 (19)	0.036 (2)	−0.0021 (16)	0.0100 (16)	0.0060 (16)
N7	0.0195 (14)	0.0251 (15)	0.0294 (16)	0.0002 (11)	0.0072 (12)	0.0037 (12)
C8	0.0207 (17)	0.0324 (19)	0.0300 (19)	0.0039 (14)	0.0024 (15)	0.0094 (15)
C11	0.0239 (17)	0.0229 (17)	0.0205 (17)	−0.0043 (14)	−0.0023 (14)	0.0037 (14)
C12	0.0271 (19)	0.033 (2)	0.033 (2)	0.0019 (15)	0.0021 (16)	0.0051 (16)
C13	0.031 (2)	0.033 (2)	0.044 (2)	0.0115 (16)	0.0010 (17)	0.0047 (17)
C14	0.043 (2)	0.0267 (19)	0.039 (2)	0.0057 (17)	−0.0032 (18)	0.0087 (17)
C15	0.033 (2)	0.0266 (18)	0.035 (2)	0.0010 (15)	0.0010 (16)	0.0059 (16)
C16	0.0247 (18)	0.0212 (17)	0.0275 (19)	0.0026 (14)	−0.0018 (15)	−0.0011 (14)
P1	0.0227 (4)	0.0234 (4)	0.0232 (5)	0.0011 (4)	0.0026 (4)	0.0045 (4)
C21	0.0227 (17)	0.0233 (17)	0.0241 (18)	0.0077 (14)	−0.0011 (14)	0.0038 (14)
C22	0.0214 (17)	0.0244 (17)	0.0279 (19)	0.0081 (14)	−0.0006 (14)	0.0070 (14)
C23	0.0246 (18)	0.037 (2)	0.035 (2)	0.0095 (15)	0.0053 (16)	0.0102 (17)
C24	0.037 (2)	0.037 (2)	0.031 (2)	0.0128 (17)	0.0066 (17)	−0.0027 (17)
C25	0.039 (2)	0.0268 (19)	0.040 (2)	0.0050 (16)	0.0053 (18)	−0.0015 (17)
C26	0.0282 (19)	0.0265 (18)	0.037 (2)	0.0016 (15)	0.0020 (16)	0.0031 (16)
C31	0.0258 (18)	0.0249 (18)	0.0243 (18)	−0.0014 (14)	0.0013 (14)	0.0071 (15)
C32	0.034 (2)	0.044 (2)	0.034 (2)	0.0090 (17)	0.0017 (17)	0.0109 (17)
C33	0.030 (2)	0.060 (3)	0.047 (3)	0.0102 (19)	−0.0033 (19)	0.023 (2)
C34	0.049 (3)	0.050 (2)	0.039 (2)	−0.006 (2)	−0.018 (2)	0.015 (2)
C35	0.060 (3)	0.034 (2)	0.030 (2)	0.0030 (19)	−0.0057 (19)	0.0008 (17)
C36	0.041 (2)	0.0298 (19)	0.030 (2)	0.0033 (16)	−0.0035 (17)	0.0039 (16)
C41	0.0269 (18)	0.0207 (17)	0.0279 (19)	0.0032 (14)	0.0048 (15)	0.0009 (14)
C42	0.0282 (19)	0.0305 (19)	0.035 (2)	0.0023 (15)	0.0011 (16)	0.0098 (16)
C43	0.0258 (19)	0.031 (2)	0.047 (2)	−0.0009 (16)	−0.0001 (17)	0.0053 (17)
C44	0.035 (2)	0.030 (2)	0.049 (2)	−0.0072 (16)	0.0095 (18)	0.0071 (18)
C45	0.048 (2)	0.034 (2)	0.043 (2)	−0.0028 (18)	−0.0022 (19)	0.0145 (18)
C46	0.035 (2)	0.034 (2)	0.033 (2)	−0.0031 (16)	−0.0018 (17)	0.0084 (17)
C51	0.064 (3)	0.064 (3)	0.053 (3)	0.013 (2)	−0.004 (2)	−0.006 (2)
Cl3	0.0649 (14)	0.0762 (16)	0.0465 (10)	0.0169 (12)	−0.0063 (8)	0.0052 (9)
Cl4	0.0406 (10)	0.120 (3)	0.0799 (18)	−0.0128 (13)	0.0074 (11)	−0.0469 (16)
C51'	0.064 (3)	0.064 (3)	0.053 (3)	0.013 (2)	−0.004 (2)	−0.006 (2)
Cl3'	0.079 (8)	0.090 (10)	0.083 (7)	0.027 (8)	−0.006 (6)	−0.009 (7)
Cl4'	0.065 (9)	0.140 (15)	0.083 (8)	−0.005 (10)	0.014 (6)	0.038 (11)

### Geometric parameters (Å, º)

**Table:**

Ru1—C1	1.860 (4)	C23—H23	0.9400
Ru1—C2	1.932 (4)	C24—C25	1.381 (5)
Ru1—C3	2.065 (3)	C24—H24	0.9400
Ru1—C11	2.129 (3)	C25—C26	1.386 (5)
Ru1—P1	2.4265 (9)	C25—H25	0.9400
Ru1—Cl1	2.4737 (9)	C26—H26	0.9400
C1—O1	1.127 (4)	C31—C36	1.381 (5)
C2—O2	1.135 (4)	C31—C32	1.386 (4)
C3—N7	1.345 (4)	C32—C33	1.391 (5)
C3—N4	1.364 (4)	C32—H32	0.9400
N4—C5	1.382 (4)	C33—C34	1.373 (5)
N4—C16	1.432 (4)	C33—H33	0.9400
C5—C6	1.340 (5)	C34—C35	1.382 (5)
C5—H5	0.9400	C34—H34	0.9400
C6—N7	1.387 (4)	C35—C36	1.386 (5)
C6—H6	0.9400	C35—H35	0.9400
N7—C8	1.465 (4)	C36—H36	0.9400
C8—C22	1.518 (4)	C41—C42	1.394 (4)
C8—H8A	0.9800	C41—C46	1.396 (4)
C8—H8B	0.9800	C42—C43	1.378 (4)
C11—C12	1.379 (4)	C42—H42	0.9400
C11—C16	1.404 (4)	C43—C44	1.384 (5)
C12—C13	1.391 (5)	C43—H43	0.9400
C12—H12	0.9400	C44—C45	1.373 (5)
C13—C14	1.374 (5)	C44—H44	0.9400
C13—H13	0.9400	C45—C46	1.385 (5)
C14—C15	1.391 (5)	C45—H45	0.9400
C14—H14	0.9400	C46—H46	0.9400
C15—C16	1.387 (4)	C51—Cl4	1.728 (10)
C15—H15	0.9400	C51—Cl3	1.744 (7)
P1—C31	1.834 (3)	C51—H51A	0.9800
P1—C41	1.836 (3)	C51—H51B	0.9800
P1—C21	1.841 (3)	C51'—Cl4'	1.727 (11)
C21—C26	1.396 (4)	C51'—Cl3'	1.744 (8)
C21—C22	1.409 (4)	C51'—H51C	0.9800
C22—C23	1.383 (4)	C51'—H51D	0.9800
C23—C24	1.380 (5)		
			
C1—Ru1—C2	91.19 (14)	C22—C21—P1	121.2 (2)
C1—Ru1—C3	87.39 (13)	C23—C22—C21	119.4 (3)
C2—Ru1—C3	171.22 (13)	C23—C22—C8	118.1 (3)
C1—Ru1—C11	89.85 (13)	C21—C22—C8	122.5 (3)
C2—Ru1—C11	93.55 (13)	C24—C23—C22	121.8 (3)
C3—Ru1—C11	77.79 (12)	C24—C23—H23	119.1
C1—Ru1—P1	95.28 (10)	C22—C23—H23	119.1
C2—Ru1—P1	93.06 (10)	C23—C24—C25	119.3 (3)
C3—Ru1—P1	95.70 (9)	C23—C24—H24	120.3
C11—Ru1—P1	171.55 (9)	C25—C24—H24	120.3
C1—Ru1—Cl1	174.32 (10)	C24—C25—C26	119.8 (3)
C2—Ru1—Cl1	92.23 (10)	C24—C25—H25	120.1
C3—Ru1—Cl1	88.54 (9)	C26—C25—H25	120.1
C11—Ru1—Cl1	85.41 (8)	C25—C26—C21	121.6 (3)
P1—Ru1—Cl1	89.07 (3)	C25—C26—H26	119.2
O1—C1—Ru1	177.0 (3)	C21—C26—H26	119.2
O2—C2—Ru1	175.9 (3)	C36—C31—C32	118.8 (3)
N7—C3—N4	104.1 (3)	C36—C31—P1	118.1 (3)
N7—C3—Ru1	139.1 (2)	C32—C31—P1	123.1 (3)
N4—C3—Ru1	116.6 (2)	C31—C32—C33	120.5 (4)
C3—N4—C5	111.4 (3)	C31—C32—H32	119.8
C3—N4—C16	116.8 (3)	C33—C32—H32	119.8
C5—N4—C16	131.4 (3)	C34—C33—C32	120.3 (4)
C6—C5—N4	106.2 (3)	C34—C33—H33	119.8
C6—C5—H5	126.9	C32—C33—H33	119.8
N4—C5—H5	126.9	C33—C34—C35	119.3 (3)
C5—C6—N7	107.3 (3)	C33—C34—H34	120.3
C5—C6—H6	126.4	C35—C34—H34	120.3
N7—C6—H6	126.4	C34—C35—C36	120.5 (4)
C3—N7—C6	111.1 (3)	C34—C35—H35	119.8
C3—N7—C8	123.5 (3)	C36—C35—H35	119.8
C6—N7—C8	125.3 (3)	C31—C36—C35	120.5 (3)
N7—C8—C22	112.9 (2)	C31—C36—H36	119.7
N7—C8—H8A	109.0	C35—C36—H36	119.7
C22—C8—H8A	109.0	C42—C41—C46	118.6 (3)
N7—C8—H8B	109.0	C42—C41—P1	120.5 (2)
C22—C8—H8B	109.0	C46—C41—P1	120.8 (2)
H8A—C8—H8B	107.8	C43—C42—C41	121.0 (3)
C12—C11—C16	115.6 (3)	C43—C42—H42	119.5
C12—C11—Ru1	130.3 (2)	C41—C42—H42	119.5
C16—C11—Ru1	114.0 (2)	C42—C43—C44	119.6 (3)
C11—C12—C13	122.0 (3)	C42—C43—H43	120.2
C11—C12—H12	119.0	C44—C43—H43	120.2
C13—C12—H12	119.0	C45—C44—C43	120.2 (3)
C14—C13—C12	120.4 (3)	C45—C44—H44	119.9
C14—C13—H13	119.8	C43—C44—H44	119.9
C12—C13—H13	119.8	C44—C45—C46	120.6 (3)
C13—C14—C15	120.2 (3)	C44—C45—H45	119.7
C13—C14—H14	119.9	C46—C45—H45	119.7
C15—C14—H14	119.9	C45—C46—C41	120.0 (3)
C16—C15—C14	117.7 (3)	C45—C46—H46	120.0
C16—C15—H15	121.2	C41—C46—H46	120.0
C14—C15—H15	121.2	Cl4—C51—Cl3	111.5 (8)
C15—C16—C11	124.0 (3)	Cl4—C51—H51A	109.3
C15—C16—N4	121.3 (3)	Cl3—C51—H51A	109.3
C11—C16—N4	114.7 (3)	Cl4—C51—H51B	109.3
C31—P1—C41	101.78 (14)	Cl3—C51—H51B	109.3
C31—P1—C21	104.91 (14)	H51A—C51—H51B	108.0
C41—P1—C21	102.61 (14)	Cl4'—C51'—Cl3'	111.4 (9)
C31—P1—Ru1	114.52 (10)	Cl4'—C51'—H51C	109.4
C41—P1—Ru1	116.91 (10)	Cl3'—C51'—H51C	109.4
C21—P1—Ru1	114.40 (10)	Cl4'—C51'—H51D	109.3
C26—C21—C22	118.1 (3)	Cl3'—C51'—H51D	109.3
C26—C21—P1	120.6 (2)	H51C—C51'—H51D	108.0
			
C1—Ru1—C3—N7	−80.2 (4)	Cl1—Ru1—P1—C41	63.93 (12)
C11—Ru1—C3—N7	−170.6 (4)	C1—Ru1—P1—C21	120.34 (15)
P1—Ru1—C3—N7	14.8 (3)	C2—Ru1—P1—C21	−148.19 (14)
Cl1—Ru1—C3—N7	103.8 (3)	C3—Ru1—P1—C21	32.44 (14)
C1—Ru1—C3—N4	94.1 (2)	Cl1—Ru1—P1—C21	−56.00 (11)
C11—Ru1—C3—N4	3.7 (2)	C31—P1—C21—C26	−111.9 (3)
P1—Ru1—C3—N4	−170.9 (2)	C41—P1—C21—C26	−5.9 (3)
Cl1—Ru1—C3—N4	−81.9 (2)	Ru1—P1—C21—C26	121.7 (2)
N7—C3—N4—C5	−0.2 (3)	C31—P1—C21—C22	71.3 (3)
Ru1—C3—N4—C5	−176.4 (2)	C41—P1—C21—C22	177.3 (2)
N7—C3—N4—C16	172.8 (2)	Ru1—P1—C21—C22	−55.0 (3)
Ru1—C3—N4—C16	−3.3 (3)	C26—C21—C22—C23	0.0 (4)
C3—N4—C5—C6	0.1 (4)	P1—C21—C22—C23	176.8 (2)
C16—N4—C5—C6	−171.7 (3)	C26—C21—C22—C8	178.1 (3)
N4—C5—C6—N7	0.1 (4)	P1—C21—C22—C8	−5.1 (4)
N4—C3—N7—C6	0.3 (3)	N7—C8—C22—C23	−92.6 (3)
Ru1—C3—N7—C6	175.1 (3)	N7—C8—C22—C21	89.3 (4)
N4—C3—N7—C8	−175.0 (3)	C21—C22—C23—C24	−1.0 (5)
Ru1—C3—N7—C8	−0.2 (5)	C8—C22—C23—C24	−179.2 (3)
C5—C6—N7—C3	−0.3 (4)	C22—C23—C24—C25	1.8 (5)
C5—C6—N7—C8	174.9 (3)	C23—C24—C25—C26	−1.5 (5)
C3—N7—C8—C22	−74.5 (4)	C24—C25—C26—C21	0.6 (5)
C6—N7—C8—C22	110.9 (3)	C22—C21—C26—C25	0.2 (5)
C1—Ru1—C11—C12	91.5 (3)	P1—C21—C26—C25	−176.6 (3)
C2—Ru1—C11—C12	0.3 (3)	C41—P1—C31—C36	76.7 (3)
C3—Ru1—C11—C12	178.9 (3)	C21—P1—C31—C36	−176.7 (2)
Cl1—Ru1—C11—C12	−91.6 (3)	Ru1—P1—C31—C36	−50.4 (3)
C1—Ru1—C11—C16	−90.8 (2)	C41—P1—C31—C32	−102.9 (3)
C2—Ru1—C11—C16	178.0 (2)	C21—P1—C31—C32	3.8 (3)
C3—Ru1—C11—C16	−3.5 (2)	Ru1—P1—C31—C32	130.0 (2)
Cl1—Ru1—C11—C16	86.0 (2)	C36—C31—C32—C33	2.4 (5)
C16—C11—C12—C13	1.5 (5)	P1—C31—C32—C33	−178.0 (3)
Ru1—C11—C12—C13	179.1 (2)	C31—C32—C33—C34	−1.0 (5)
C11—C12—C13—C14	−0.7 (5)	C32—C33—C34—C35	−1.4 (6)
C12—C13—C14—C15	−0.5 (5)	C33—C34—C35—C36	2.3 (5)
C13—C14—C15—C16	0.9 (5)	C32—C31—C36—C35	−1.6 (5)
C14—C15—C16—C11	−0.1 (5)	P1—C31—C36—C35	178.8 (3)
C14—C15—C16—N4	177.8 (3)	C34—C35—C36—C31	−0.8 (5)
C12—C11—C16—C15	−1.1 (5)	C31—P1—C41—C42	−167.7 (3)
Ru1—C11—C16—C15	−179.1 (2)	C21—P1—C41—C42	83.9 (3)
C12—C11—C16—N4	−179.1 (3)	Ru1—P1—C41—C42	−42.1 (3)
Ru1—C11—C16—N4	2.9 (3)	C31—P1—C41—C46	15.8 (3)
C3—N4—C16—C15	−177.9 (3)	C21—P1—C41—C46	−92.6 (3)
C5—N4—C16—C15	−6.5 (5)	Ru1—P1—C41—C46	141.4 (2)
C3—N4—C16—C11	0.2 (4)	C46—C41—C42—C43	0.6 (5)
C5—N4—C16—C11	171.6 (3)	P1—C41—C42—C43	−176.0 (3)
C1—Ru1—P1—C31	−0.83 (16)	C41—C42—C43—C44	−1.7 (5)
C2—Ru1—P1—C31	90.64 (15)	C42—C43—C44—C45	1.6 (6)
C3—Ru1—P1—C31	−88.73 (14)	C43—C44—C45—C46	−0.4 (6)
Cl1—Ru1—P1—C31	−177.17 (12)	C44—C45—C46—C41	−0.7 (6)
C1—Ru1—P1—C41	−119.73 (16)	C42—C41—C46—C45	0.6 (5)
C2—Ru1—P1—C41	−28.25 (15)	P1—C41—C46—C45	177.2 (3)
C3—Ru1—P1—C41	152.38 (14)		
